# Comparative Analysis of Purine Alkaloids and Main Quality Components of the Three *Camellia* Species in China

**DOI:** 10.3390/foods11050627

**Published:** 2022-02-22

**Authors:** Wen Zeng, Zhen Zeng, Jie Teng, Dylan O’Neill Rothenberg, Mengzhen Zhou, Ronghui Lai, Xingfei Lai, Wenfang Zhao, Dan Li, Changyu Yan, Yahui Huang

**Affiliations:** 1 Department of Tea Sciences, College of Horticulture, South China Agricultural University, Guangzhou 510642, China; wenzeng_t@163.com (W.Z.); zzhen2004@126.com (Z.Z.); tengjie33@163.com (J.T.); dylan.rothenberg@colorado.edu (D.O.R.); xingfeilai@163.com (X.L.); 15521046764@163.com (W.Z.); 30004411@scau.edu.cn (D.L.); 2 Meizhou Academy of Agricultural Sciences, Meizhou 514021, China; zhou1756312057@163.com (M.Z.); lrhrara@163.com (R.L.); 3 Guangdong Provincial Key Laboratory of Nutraceuticals and Functional Foods, Guangzhou 510642, China

**Keywords:** wild *Camellia* species, purine alkaloid, theobromine, low-caffeine, tea products

## Abstract

Tea (*Camellia* *sinensis var. sinensis*) is a widely consumed caffeine-containing beverage, however the *Camellia* genus also includes other species, which are consumed as tea in their local growing regions. Presently, HPLC analysis assessed 126 unique *Camellia* germplasms belonging to three *Camellia* species, *C. sinensis var. pubilimba Chang* (*Csp*), *C. gymnogyna Chang* (*CgC*) and *C. crassicolumna Chang* (*CcC*). Theobromine was the predominant purine alkaloid in all species, representing over 90% of purine alkaloids in *Csp* and *CgC*, and 50% in *CcC*. Significant variability existed in purine alkaloid patterns both between and within species, and some germplasms possessed highly unique alkaloid profiles. Sensory evaluation and quality composition analysis of green tea products produced from the three *Camellia* species suggested their unsuitability for use in tea production due to their unpalatable flavor. The results of this study revealed the differences in purine alkaloids and main quality components between *Camellia* species and tea, which contributed to understand why tea, rather than other *Camellia* species, has become a popular beverage in the world after long-term artificial selection. In addition, unique alkaloid profiles suggest usefulness of these germplasm resources in future breeding of decaffeinated tea plant varieties and alkaloid metabolism research.

## 1. Introduction

Tea, second only to water in global beverage consumption, is the oldest (since 3000 BC) and most popular non-alcoholic beverage, with great economic importance and medicinal benefits. Tea is widely consumed by 3 billion people in 160 countries [1,2,3]. According to the Food and Agriculture Organization of the United Nations (https://www.fao.org/faostat/en/, accessed on 12 January 2022), China is the largest tea-producer in the world and has the most tea product types, in addition to abundant tea plant germplasm resources.

Tea plants (Sect. *Thea* (L.) Dyer) are classified into Theaceae, Genus *Camellia* Linn., and are evergreen trees or shrubs. Tea plants are classified into five species, including *C. sinensis (L.) O.* Kuntze, *C. crassicolumna* Chang (*CcC*), *C. gymnogyna* Chang (*CgC*), *C. taliensis* (W. W. Smith) Melchior and *C. tachangensis* F. S. Zhang. There are three varieties of *C. sinensis (L.) O.* Kuntze, namely *C. sinensis var. sinensis* (*Css*), *C. sinensis var. assamica* (Masters) Kitamura, and *C. sinensis var. pubilimba* Chang *(Csp)* [4,5,6]. The tea typically cultivated and consumed around the world is made from *Css*. (Figure S1) China is the origin of tea plants, with a wide variety of *Camellia* plants. As the first country in the world to use tea trees, why did our ancestors choose *Css* among the many *Camellia* plants? The leaf morphology of these *Camellia* species is similar with *Css*. What is the difference in taste? How can they be distinguished? 

However, exploratory research and sample collection of these wild tea tree species has been difficult due to their narrow and variable distribution. If knowledge of the diverse *Camellia* species germplasm can be further explored, there lies great potential to improve and optimize tea crop cultivation, and it can also help us better understand why tea, rather than other *Camellia* plants, has become a popular beverage around the world.

**Figure 1:**
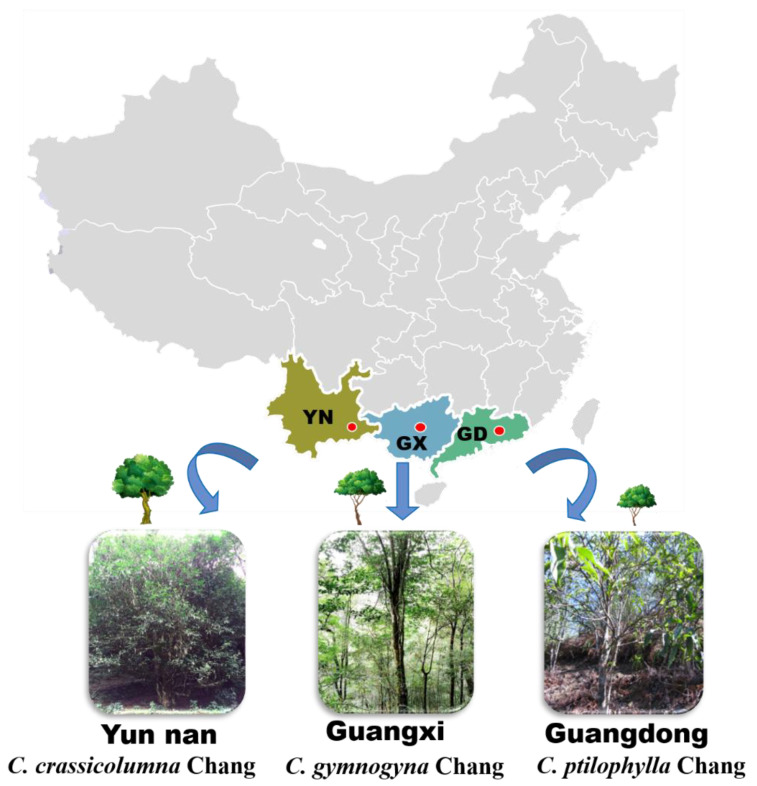
Wild germplasm resources distribution of three *Camellia* species.

Caffeine, theobromine, theophylline and theacrine are the four main purines in the category Thea, all possessing xanthine ring skeletons, but different numbers and positions of methyl groups (Figure 1). Transformation from one purine alkaloid to another depends on N-methyltransferases (NMTs). However, details regarding the metabolic network connecting the purine alkaloid compounds remain unclear [7,8,9]. In the commonly cultivated *Css*, there are three main purine alkaloids, caffeine, theobromine and theophylline, of which caffeine predominates, accounting for about 2% to 5% of tea leaf dry weight. Theobromine and caffeine both can bind to members of TAS2R bitter taste receptor family [3,10]. Caffeine is the most widely used psychoactive substance in the world [11], with many pharmacological actions, such as diuresis and central nervous system stimulation. Theobromine has similar functions but relatively low biological activity [12]. Theacrine is almost undetectable among most *Camellia* species, however is the dominant alkaloid in *Kucha (Cammelia assamica var. kucha)* [13]. Theacrine also contributes to bitterness, however exhibits opposite effects to those of caffeine and theobromine, as theacrine can be potently sedative [14]. 

Catechins account for about 80% of the total polyphenolic content in tea leaves, and 8–26% of leaf dry weight [15]. A growing number of clinical and epidemiological studies have examined the function of catechins in reducing the risk of hypertension and cardiovascular disease, and for their anti-diabetes, anti-mutation and anti-cancer properties [9,16,17,18]. Catechins are also important contributors to the bitterness and astringency of green tea, and can be divided into two types, gallated or non-gallated, depending on the presence of a galloyl moiety. Gallated catechins are the main determinants of astringency in tea and are considerably more bitter and astringent than non-gallated catechins [19,20].

As far as we know, there have been no systematic comparative studies on the alkaloid patterns and flavor of wild *Camellia* resources. 

Therefore, in order to explore the potential of unique wild *Camellia* resources in development of tea products, tea breeding materials, or potential therapeutic health agents, we collected samples from 126 accessions wild *Camellia* germplasms belonging to three species populations, *CcC*, *CgC* and *Csp*. High-performance liquid chromatography (HPLC) was used to quantitatively analyze alkaloid content. Furthermore, we processed green tea products using the raw leaf materials of the three *Camellia* species and conducted sensory evaluation and quality composition analysis on these green tea products. These results will help us to find a better way to understand the relationship between modern cultivated tea varieties and other *Camellia* species in main secondary metabolites and flavor and provided valuable references for the utilization of these particular *Camellia* resources.

## 2. Materials and Methods

### 2.1. Collection and Pretreatment of Samples

Samples from a total of 126 accessions unique wild tea germplasms were collected for analysis. Among them, 38 accessions were *CcC* collected from Wenshan County, Yunnan Province (104°14′ E, 23°23′ N, 1600 m in altitude), 36 accessions were *CgC* collected from Jinxiu County, Guangxi Province (110°18′ E, 24°13′ N, 1000 m in altitude), 52 accessions were *Csp* collected from Huizhou, Guangdong Province (113°88′ E, 23°64′ N, 700 m in altitude). The pictures of three germplasms resources are shown in Figure 1.

In the spring of 2018, 30–50 g of young shoots with one bud and two leaves were harvested from 126 individual wild tea plants. Fresh leaves of each germplasm resource were combined and analyzed as a single sample. Each sample was randomly divided into two sections. The first section was used for the quality composition analysis (leaves were fixed by microwave technology and dried at 75 °C for 32 h). In the second section, samples were processed into green tea. However, in the process of collecting wild resources, there were certain limitations due to natural conditions, such as complicated terrain and a scarcity of buds on the wild tea plants. As a result, there were not enough individual materials from each of the 126 accessions germplasm resources to be processed into 126 samples of green tea. Therefore, during the processing of fresh leaves into green tea, wild tea samples were mixed together with other leaves of the same species (*CcC*, *CgC* or *Csp*) to create three finished green tea products, one product for each wild tea species. The three green teas were produced using identical production techniques and were then utilized in sensory evaluation and quality composition analysis. All chemical composition analyses in both sections were extracted and analyzed in triplicate. 

### 2.2. Chemicals and Drugs

The standard substances, including (-)-epigallocatechin gallate (EGCG) (Purity > 97.0%), (-)-Epigallocatechin (EGC) (Purity > 95.0%), (-)-gallocatechin (GC) (Purity > 98.0%), (-)-catechin (C) (Purity > 98.0%), (-)-epicatechin (EC) (Purity > 98.0%), (-)-catechin gallate (CG), (-)-epicatechin gallate (ECG) (Purity > 98.0%), (-)-gallocatechin gallate (GCG) (Purity > 98.0%), caffeine (Purity > 98.0%), theobromine (Purity > 98.0%), theophylline (Purity > 98.0%) and theacrine (Purity > 98.0%) were purchased from Sigma-Aldrich Chemical (St. Louise, MO, USA). Methanol and acetonitrile (HPLC grade) were obtained from Tianjin Kemiou Chemical Reagent Co., Ltd. (Tianjin, China) and used as the solvents. All other chemicals and reagents used were of the highest grade available.

### 2.3. Collecting Data of Plant Morphology

The survey content includes the morphological characteristics of trees, leaves and flowers. During field investigations, morphological data was recorded such as tree type and height, and leaves were gathered for further analysis. Leaf samples were randomly selected from trees, each sample numbering between 30–40 leaves. Following data and sample collection, specimens were described in detail using the same methodologies of previous studies [5,6], and information was sorted and listed in Table 1.

**Figure 2:**
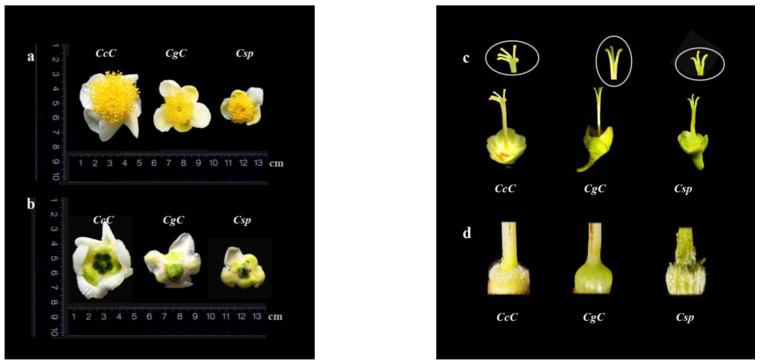

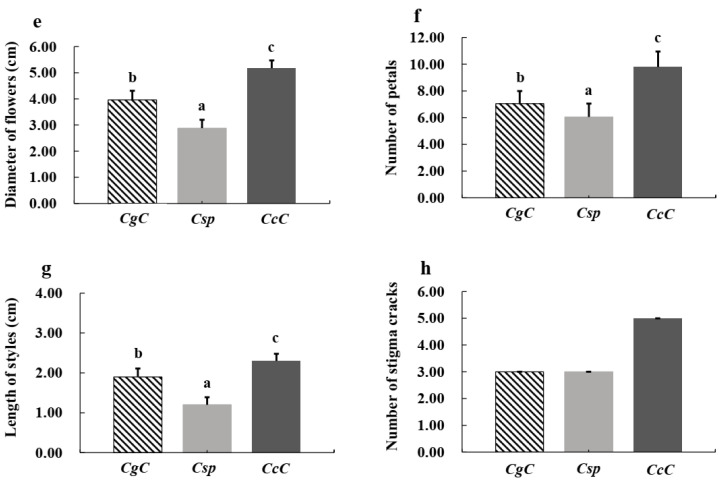
Flower Morphology of three *Camellia* species. (**a**) Front of flowers of three *Camellias* Species; (**b**) back of flowers of three *Camellia* species; (**c**) styles and stigmas of three *Camellia* species; (**d**) ovaries of three *Camellia* Species; (**e**) diameter of flowers of three *Camellia* species; (**f**) number of petals of three *Camellia* species; (**g**) length of styles of three *Camellia* species; (**h**) number of stigma cracks of three *Camellia* species; *CgC*, *C. gymnogyna Chang*; *Csp*, *C. sinensis var. pubilimba Chang*; *CcC*, *C. crassicolumna Chang*. Values are mean ± SD (*n* = 20). Different lowercase letters mean significant differences at *p* < 0.05 (Tukey’s HSD).

Observations of flower morphology were conducted from mid-November to late December 2018. Young unopened flower buds of three *Camellia* species were collected and returned to the laboratory to feed until flowering. Daily observations were made on the morphological characteristics of the blooming flowers, including flower size, petal number, style length, and number of stigma cracks. Photos taken over this period can be seen in Figure 2. 

### 2.4. Green Tea Processing Technique

Fresh leaves from three wild *Camellia* Species (*CcC*, *CgC* and *Csp*) were processed into finished loose-leaf green tea products, such as those commonly available for purchase and consumption. Processing methods were kept constant for all three teas, and were consistent with those utilized in previous reports [21]. The steps were as follows: fresh leaves were laid out to wither indoors for 2.5 h (25 °C, 70% humidity); then cooked with hot steam for 45 s; left at room temperature for 15 min; rolled mechanically for 20 min; and finally dried using two subsequent drying treatments—110 °C for 25 min, followed by 80 °C for 2.5 h. Green tea products were bagged and sealed for sensory evaluation.

### 2.5. Analysis of Purine Alkaloids in Tea Samples

The contents of purine alkaloids were determined by high-performance liquid chromatography (HPLC). 

After harvest, the fresh leaves of all 126 samples were fixed by microwave technology and dried at 75 °C for 24 h by a 1200 kW drying machine (Shanghai YuejinMedical Machine Co., Shanghai, China). A total of 0.5 g of dried and fully crushed tea sample was added to distilled water (70 mL, 100 °C), extracted for 45 min, stirred 4–5 times (every 10 min), and filtered while hot through 0.45 μm filter paper. Filtrate was transferred into a 100 mL volumetric flask, and distilld water was added until 100 mL. Finally, 1 mL pipette was taken from 100 mL volumetric flask and added to 24 mL distilled water to form a total volume of 25 mL. Samples were then filtered through a 0.22 μm membrane before analysis.

Chromatographic conditions were set as follows: HPLC (Agilent 1200, Agilent Technologies, Santa Clara, CA, USA) column used was an Agilent Eclipse XDB-C18 column (4.6 × 150 mm, 5 μm) maintained at 38 °C. The sample injection volume was 10 μL. The mobile phases were 0.1% (*v*/*v*) trifluoroacetic acid in double-distilled water (eluent A) and methanol (eluent B).

HPLC conditions for the detection of purine alkaloids were set according to our previous research [22].

### 2.6. Analysis of Catechins in Green Tea Samples

The sample extraction method for catechin detection in green tea samples was the same as that mentioned in 2.5, and chromatographic conditions were the same. HPLC conditions for catechin detection are shown in Figure S2. 

Substances were identified through comparison of retention times with those of the reference standards. Calibration curves for quantification were obtained by plotting known concentrations of standards and peak areas. The regression equations and correlation coefficients of 11 standards are summarized in the Supporting Data (Tables S1 and S2). Peak times of catechins and purine alkaloids are shown in Figure S2.

### 2.7. Analysis of Total Amino Acids in Green Tea Samples

According to the standards of GB/T 8314-2002, 1.5 g ground tea sample (accurate to 0.001 g) was added to distilled water (225 mL, 100 °C), extracted with boiling water for 45 min, stirred 2–3 times, and filtered while hot using suction filtration. Filtrate was transferred into a 250 mL volumetric flask, with distilled water added until full. Pipette of 1 mL filtrate was mixed with 0.5 mL of pH 8.0 buffer and 0.5 mL of ninhydrin as a color developer, heated in boiling water for 15 min, then added to distilled water to fix the volume at 25 mL. A blank control sample was made using distilled water instead of the sample solution. The thickness of the cuvette was 1.0 cm, and the absorbance was measured at 570 nm wavelength. The data was recorded and results calculated according to the standard curve function. All tests were completed in triplicate. 

### 2.8. Sensory Evaluation

A total of 20 trained participants were recruited for sensory evaluation of the three green tea infusions (11 women, 9 men, ages 25 to 49). Panelists were not provided with information regarding the tea samples prior to evaluation; however, detailed evaluation protocol was given in order to ensure uniform evaluation standards among participants. Sensory evaluation comprised two parts: taste and overall acceptability. In accordance with previous studies on green tea taste [20,23,24], the determinants of taste quality were divided into four elements: bitterness, astringency, sweetness and umami. The evaluators were instructed to assign scores (1–5) to characterize each element of taste [25], with the number value increasing with perceived intensity of taste element (i.e., 1 = “very weak”, 3 = “medium”, 5 = “very strong”). The same scoring method was used to evaluate overall acceptability, with 1 indicating “unacceptable,” 3 indicating “generally acceptable,” and 5 indicating “very enjoyable.”

Preparation of green tea infusions for evaluation proceeded as follows: loose-leaf green tea (3 g) was infused with boiling distilled water (100 mL) for 5 min in ceramic tea-tasting infusion cups, then poured out into bowls for consumption. The sensory evaluation of green tea solution refers to the previous methods [26].

### 2.9. Statistical Analysis of Data

Data analysis was performed using Excel (2016) and SPSS (19.0, SPSS Inc., Chicago, IL, USA). One-way analysis of variance (ANOVA) was performed, followed by a least significant difference (LSD) test and Duncan’s test. *p* < 0.05 was considered a statistically significant difference. Principal component analysis aims to use the idea of dimensionality reduction to transform multiple indicators into a few comprehensive indicators so that they reflect the main characteristics of the object. Compute the eigenvectors of the covariance matrix, project the data into the space represented by the eigenvectors, and pick the least number of dimensions to summarize the most important features. Principal component analysis was performed with SPSS, and Past (3.20) was used for plotting.

## 3. Results and Discussion

### 3.1. Morphological Characteristics of Three Camellia Species

With reference to the previous studies on classification of *Camellia* plants [4,5,6], we investigated the morphology of the three wild *Camellia* species, as detailed in the Introduction.

At present, the classification of angiosperms is mainly based on morphological data [27]. Among the morphological characteristics of flowers, fruits, roots, stems and leaves of angiosperms, floral morphology is particularly important [28]. Similarly, in the classification of *Camellia*, flowers play a decisive role [5,6]. The value of floral features towards plant classification relates to their relative morphological stability and thus their reliability as a species indicator. In contrast, the morphology of trees and leaves show large variations within contrasting environments.

Distributed in different regions, the growth environment of the three wild *Camellia* species vary widely. Therefore, for our morphological investigation, flowers were selected as our primary objects of observation, and some data of leaves and tree types were also recorded. Table 1 and Table 2 summarize the main morphological characteristics of three *Camellia* species in tree type, leaves and flowers. Photographs of tree and floral morphology are shown in Figure 1 and Figure 2. Observations were made regarding the flowers of three species (*n* = 20), including flower diameter, number of petals, length of the style and number of stigma cracks (Figure 2).

*CcC* had the largest flower diameter, 4.8–5.5 cm, and *Csp* had the smallest, 2.3–3.4 cm (Table 1 and Figure 2a,b). The styles of *CgC* and *Csp* were 3-parted, while *CcC* was 5-parted (Figure 2c). The ovaries of all three were spherical, however the outer ovary of *CcC* and *Csp* were covered with white hairs, while that of *CgC* was smooth and glabrous (Figure 2d).

All tea plants belonged to evergreen trees or shrubs, possessing white flowers with 5–15 petals, ovaries with or without white hair, and the number of cracks in stigma is (2) 3–5 (7) [5]. Among tea plants, more petals indicate older evolutionary history than less petals, while 5-parted styles indicate older evolutionary history than 3-parted [5,6]. Among the three *Camellia* variants, *CcC* showed indications of being the most primitive, *CgC* appeared to be in the middle, while *Csp* appeared to be the most recently evolved. Flowers of *Css* had 5-parted styles, ovaries with white hair, petals 5–12 [5]; *Csp* and *Css* are different variants of the same species (*C. sinensis* (*L.*) *O.* Kuntze), so the flower morphology of *Csp* most closely resembled that of cultivated tea plants (*Css*).

### 3.2. Patterns of Purine Alkaloids in Three Camellia Species

**Figure 3:**
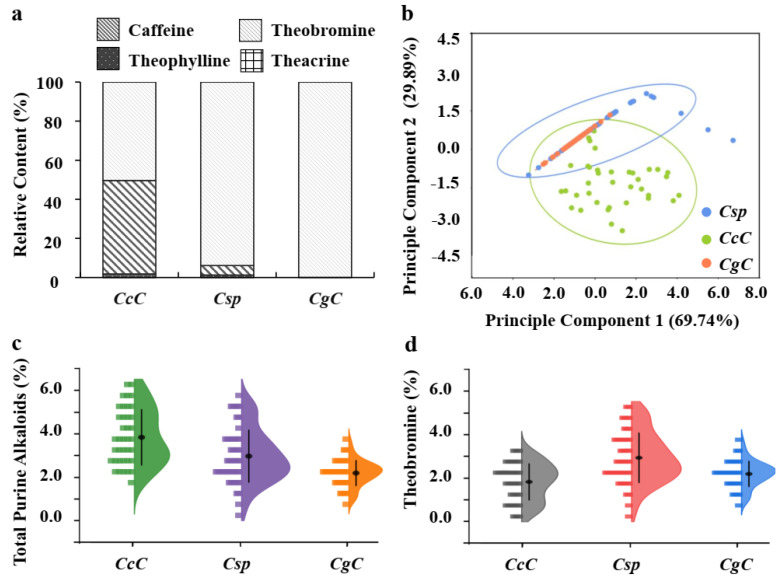
Distribution of purinealkaloids in three *Camellia* species. (**a**) Relative contents of four purine alkaloids; (**b**) principal component analysis (PCA) of four purine alkaloids derived from 126 accessions wild tea tree samples of three *Camellia* species; (**c**) percentage of theobromine in three *Camellia* species (percentage of dry weight); (**d**) percentage of total purine alkaloids in three *Camellia* species (percentage of dry weight). *CgC*—C. gymnogyna Chang; *Csp*—C. sinensis var. pubilimba Chang; *CcC*—*C. crassicolumna Chang*.

Theobromine was the predominant purine alkaloid among the three *Camellia* species in this survey (Table 3, Figure 3a), however overall alkaloid patterns among the three species varied. All four types of purine alkaloids were detected in *CcC*, three types were detected in *Csp*, and only one alkaloid was detected in *CgC*, theobromine (Table 3). The average caffeine content of *CcC* was higher than the other two species.

Principal component analysis (PCA) was performed based on four alkaloid types of 126 accessions wild tea tree samples of three Camellia species. Table 4 shows three principal components. Principal component 1 (PC1) and principal component 2 (PC2) together account for 99.63% of the total difference. PC1 is mainly affected by the total amount of purine alkaloids, while theobromine dominates PC2. The spatial distribution of 126 Camellia plants related to PC1 and PC2 is shown in Figure 3b, with different species distinguished by color. PCA analysis can distinguish *CsC* from the other two species, but the difference between *Csp* and *CgC* is not obvious. It is speculated that both *Csp* and *CgC* take theobromine as the main alkaloid, and the total alkaloids of the two species are similar.

We assessed the distribution of purine alkaloids of various germplasms within the same *Camellia* specie in order to find intraspecies variation. In *Csp*, theobromine was the most abundant (90%) (Figure 3a), several germplasms belonging to *Csp* also contained trace amounts of theophylline, and one unique germplasm contained high levels of caffeine (Table 5). In *CcC*, caffeine and theobromine accounted for more than 95% of the total purine alkaloids (Figure 3a), representing 1.93% and 1.83% of total tea leaf dry weight, respectively (Table 3); however, some unique germplasms also contain trace amounts of theophylline and theacrine (Table 5). In short, the variation in alkaloid profile not only existed among species, but also within species. Theacrine is mainly detected from Kucha (C. assamica var. kucha) [13], and its content is very low in other *Camellia* species and some other plants (e.g., *Coffea genus*, *Herrania* and *Theobroma* spp.) [29]. Theacrine and caffeine are the main purine alkaloids of Kucha [30]. 

In the cultivated tea plant (*Css*), there is one single pattern of alkaloids—caffeine being predominant. From the perspective of the three *Camellia* species resources of this survey, the alkaloid profiles were highly variable among species, but also variable within the same species. Theobromine, rather than caffeine, was the predominant alkaloid in the three *Camellia* species. Compared with their wild relatives, cultivated tea plants often lose diversity under the influence of long-term human selection.

### 3.3. Variation of Theobromine Content in Three Camellia Species

Theobromine accounted for over 90% of the total alkaloids in *CgC* and *Csp* (Figure 3a), yet its content was variable across samples. Comparing the variation of theobromine content among different species, the variation range of *Csp* was the widest (0.38–5.25%), and *CgC* was the narrowest (0.97–3.64%). In terms of average theobromine content, *Csp* was the highest (2.93 ± 0.16), *CcC* was the lowest (1.83 ± 0.14). Regarding total alkaloid content, *CcC* (3.83% ± 1.29) was the highest, and *CgC* is the lowest (2.19 ± 0.60) (Table 3). The distribution and variation of theobromine and total purine alkaloid contents of three *Camellia* species are shown in Figure 3c,d.

The greatest difference between the three *Camellia* species investigated in the present study and the commonly cultivated *Css* is that *Css* does not have theobromine as the most abundant purine alkaloid. Furthermore, in our study the theobromine content, range of variation, and representative proportion of total alkaloids were different among the three species, and some unique germplasms within a species possessed irregular alkaloid profiles (Table 5). Previous studies have shown that *C. ptilophylla* Chang (*Csp*) is generally considered to be a low- or no-caffeine tea plant [31], commonly referred to as called ‘cocoa tea’, as theobromine is the primary alkaloid in cocoa. However, in the current investigation, we found that other *Camellia* species than *Csp* also possessed theobromine as the predominant alkaloid.

### 3.4. Unique Germplasm Resources Discovered in Three Camellia Species

1. Unique germplasm resources of *C. sinensis var. pubilimba Chang* (*Csp*)

To the best of our knowledge, the current study is the first to report that theophylline exists in *C. ptilophylla* Chang (*Csp*) (Table 5). Since its discovery in China in 1988 [32], *Csp* has been considered a novelty tea beverage due to its limited or entire lack of caffeine. Theobromine replaces caffeine as the primary alkaloid in *Csp*, giving it the name cocoa tea [31]. In the current study, we found one *Csp* germplasm with high caffeine content (3.09 ± 0.01), which is a unique finding, albeit one that can be supported by previous findings [31].

2. Special germplasm resources *C. crassicolumna Chang* (*CcC*)

According to our research data, the *CcC* sample population included varied patterns of alkaloids, including low caffeine or no caffeine (Table 5), which contradicts the results of some previous studies [33,34,35]. In addition to low caffeine contents, some unique *CcC* germplasms contained trace amounts of theacrine or theophylline, or contained high caffeine (3.63 ± 0.03) (Table 5). *CcC* germplasms possessing these unique characteristics have not been previously reported, and they help to paint a more nuanced picture of the *C. crassicolumna* Chang species.

3. Special germplasm resources *C. gymnogyna Chang (CgC)*

Our data showed that the population of *C. gymnogyna* Chang sampled in this study was a natural caffeine-free tea plant resource, as no caffeine was able to be detected in any *CgC* samples. Our previous study found that a population of *CgC* contained theobromine, caffeine and theophylline, with theobromine being the most abundant [22]. However, the results of the current study vary from those previously reported, as only theobromine was able to be detected in this *CgC* population. 

### 3.5. Quality Composition Analysis and Sensory Evaluation of Green Tea 

**Figure 4:**
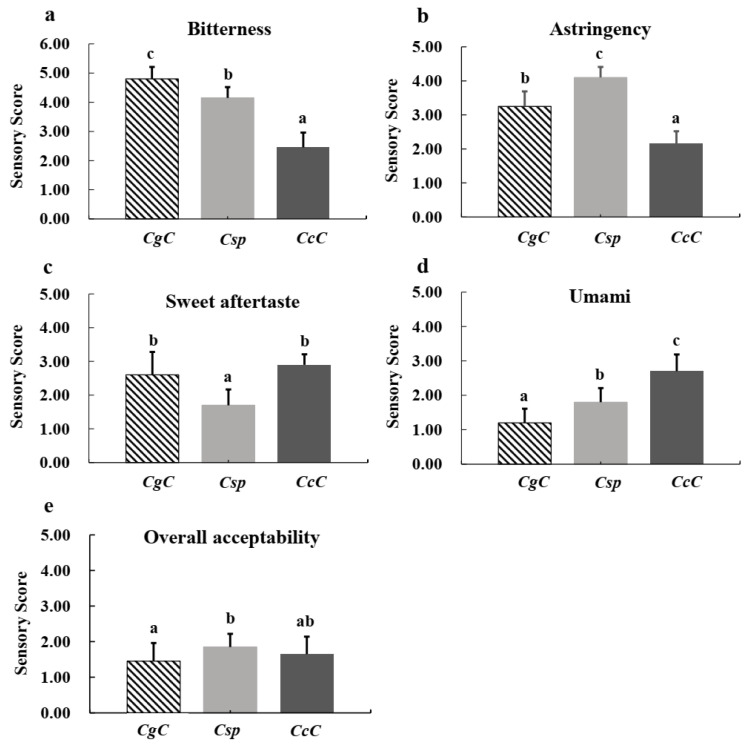
Sensory evaluation results of green tea infusions from three *Camellia* species. (**a**) Sensory scores of bitterness of green tea infusions from three *Camellia* species; (**b**) sensory scores of astringency of green tea infusions from three *Camellia* species; (**c**) sensory scores of sweet aftertaste of green tea infusions from three *Camellia* species; (**d**) sensory scores of umami of green tea infusions from three *Camellia* species; (**e**) sensory scores of overall acceptability of green tea infusions from three *Camellia* species. *CgC*—C. *gymnogyna Chang*; *Csp*—*C. sinensis var. pubilimba Chang*; *CcC*—*C. crassicolumna Chang*; Values are mean ± SD (*n* = 20). Different lowercase letters mean significant differences at *p* < 0.05 (Tukey’s HSD).

Three finished green tea products produced from *CcC*, *CgC*, or *Csp* were evaluated by 20 highly-trained panelists. The teas were judged based on five points: bitterness, astringency, sweet aftertaste, umami and overall acceptability (Figure 4). Assessing the results of the evaluation, we found that the intensity of bitterness of *CgC* green tea was the strongest, *CcC* green tea attained the highest score for sweet aftertaste. *Csp* green tea possessed the strongest astringency, while the umami intensity of all three green teas were below “normal”. In terms of overall acceptability, all three green tea products were between “unacceptable” and “generally acceptable”.

Catechins, purine alkaloids and amino acids (primarily theanine) are the three main secondary metabolites in tea plant (*Camellia sinensis*) and play an important role in flavor and health benefits. Catechins, purine alkaloids and total amino acid content of three green tea products, are shown in Table 6. Four types of catechins were detected in *CcC* green tea, seven types in *CgC*, and eight types in *Csp*. The eight types of catechins found in *Csp* were the same eight present in the commonly consumed *Css* variant. *Csp* and *Css* are considered to be different variants of the same species, which can explain how they have similar catechin profiles. Similarly, *Kucha* and *Css* are also different variants of the same species, and seven types of catechins were detected from *Kucha* in previous studies [29].

In the process of cultivation, domestication and evolution for thousands of years, under the pressure of farmers and environmental selection, modern cultivated tea varieties contain high catechins (primarily ester-type catechins). Ester-type catechins account for 75% of the total catechins [36]. Among the three green tea products, the percentage of ester-type catechins in total catechins of *Csp* was 60.23%, *CcC* and *CgC* were 42.14% and 36.33% (Table 6), respectively. According to previous reports, about 50% in *Kucha* [29].

*Csp* green tea had the highest total catechin content (13.25 ± 0.04), compared with *CcC* (4.01 ± 0.02) and *CgC* (5.12 ± 0.01). Caffeine was detected in *CcC* (1.56 ± 0.01) and *Csp* (0.07 ± 0.01), while our assessment of total amino acids showed that *CcC* was highest (1.31 ± 0.03), while *CgC* was the lowest (0.75 ± 0.01).

EGCG and ECG are highly correlated with the degree of bitterness in green tea, while esterified catechins stand as the main source of astringency in a tea infusion [19,20], and the concentration of ECG and flavonol glycosides is highly correlated with astringency [20]. The total contents of EGCG and ECG found in the three green tea products, from high to low, were *Csp* (2.09 ± 0.02), *CgC* (1.84 ± 0.03), *CcC* (1.69 ± 0.02) (Table 6), which correlated with the bitterness intensity, from strong to weak, *CgC*, *Csp*, *CcC* (Figure 4), and astringency intensity from strong to weak, namely, *Csp*, *CgC* and *CcC* (Figure 4). Although there was a positive relationship between EGCG/ECG contents and bitterness/astringency, the presence of these catechin compounds were likely not the sole determinants of the ultimate bitterness/astringency detected by panelists. Previous studies have shown that interactions take place between taste substances, exerting both synergistic and counteractive effects; EGCG and caffeine can enhance the astringency and bitterness of each other, theanine can reduce the astringency of EGCG solutions, and the astringency of EGCG can be weakened by pectin [20,37,38,39].

The amino acid content of *Csp* was higher than *CgC*, which may have contributed to the lower bitterness intensity of *Csp* than *CgC*, even though the total contents of EGCG and ECG in *Csp* were higher than *CgC*. Amino acids can exhibit various taste characteristics in tea, including umami, sweet, sour, or even bitter, the latter caused by the hydrophobic effect of amino acid side chains [40]. 

In a green tea infusion, EGC and EC contribute to a sweet aftertaste, however the intensity of the sweet aftertaste is affected by other characteristics of the infusion. For example, astringency significantly inhibits the development of sweet aftertaste, however bitterness does not, while an umami flavor can slightly enhance a sweet aftertaste [26]. Measuring the total contents of EGC and EC (Table 6), *CcC* was the highest (2.32 ± 0.03), which coincided with it possessing the highest intensity of sweet aftertaste. Although the total amount of EGC and EC of *CcC* (2.08 ± 0.01) was similar to *CgC* (2.32 ± 0.03), the astringency of *CgC* was stronger than *CcC*, which likely inhibited the sweet aftertaste characteristic, leading to the relatively weak sweet aftertaste of *CgC* compared *CcC*. Along the same reasoning, *Csp* scored both the highest intensity of astringency and the weakest intensity of sweet aftertaste.

Amino acids are the primary contributors to umami flavor in green tea, and glutamic acid and theanine collectively account for approximately two-thirds of the total amino acids in green tea [25]. L-theanine, gallic acid, succinic acid, and theophylline have also been found to enhance the umami flavor of L-glutamate in green tea, while caffeine was shown to enhance the umami intensity of theanine [41]. The umami intensity, from strong to weak, was: *CcC*, *Csp*, *CgC* (Figure 4). As shown in Table 6 *CgC* possessed the lowest content of amino acids (0.75 ± 0.02), while *Csp* (1.06 ± 0.05) was similar to *CcC* (1.31 ± 0.06). However, caffeine in *CcC* (1.56 ± 0.01) was significantly higher than *Csp* (0.07 ± 0.01), which may have helped to enhance the umami intensity of *CcC*, leading to its relatively high umami intensity compared to *Csp*. In summary, the flavor of tea is a complex multifactorial topic, pertaining not only to the type and content of individual compounds, but also the interactions among substances in the solution environment.

The general acceptability of *Csp* was rated slightly higher than *CcC* and *CgC*, however the ratings of all three green tea products were below “generally acceptable” (Figure 4). It is noteworthy that the taste of green tea processed by all three wild *Camellia* species in this study was not satisfactory. 

*Camellia* species or varieties have the same or similar ancestors as modern cultivated tea varieties, but they show great differences from modern cultivated tea in the main secondary metabolites through farmers and environmental selection history. The accumulation mechanism and pathway of these main secondary metabolites have been studied thoroughly [42,43]. A series of studies showed that tandem duplication and PPC-WGD (Polemonioids-Primuloids-Core Ericales-Whole-genome triplication) have proved to be important contributions to the accumulation of these special metabolites. For example, the researchers revealed that the *NMT* genes are involved in the metabolism of caffeine and the *SCPL* genes are involved in the metabolism of catechins, which were expanded by tandem duplications in the tea plant genome [2,44]. On the one hand, the molecular mechanism of biosynthesis and regulation underlying these secondary metabolites has been widely explored. On the other hand, from a macroscopic perspective, we characterized the main metabolites in those *Camellia* species, and explored their sensory evaluation characteristics as beverages, which provide a new insight for comprehensive understanding of the relationship between modern cultivated tea varieties and other wild *Camellia* species in components and evolution.

In China, the leaves of many *Camellia* plants are normally referred to as ‘wild tea trees’ by local peoples, and can also be consumed as tea, including *CcC*, *Csp* and *CgC*. [41]. However, combining sensory evaluation and quality composition analysis, we found that the types and contents of catechins, purine alkaloids, and total amino acids in these three *Camellia* species are significantly different from tea, and their taste characteristics make them unsuitable for larger-scale commercialization and widespread adoption as a tea beverage.

In addition, our investigation also found that, in China, most wild *Camellia* species are distributed in areas inhabited by ethnic minorities. In terms of leaf morphology, these wild *Camellia* species are very similar to *Css*, but the taste is very different. In areas where *Css* and other *Camellia* species coexist, ethnic minorities can distinguish *Css* from *Camellia* species by observing the leaves or directly chewing fresh leaves to feel its taste. Maybe our ancestors distinguished *Css* from *Camellia* plants in a similar way. At the same time, we found that the natives would directly use *Css* to make tea products for drinking. For other *Camellia* plants, it is rarely drank directly, but processed into tea products and stored for several years before drinking to make it taste better.

## 4. Conclusions

In the present study we collected preliminary plant morphological data of three *Camellia* species, *Csp*, *CcC*, and *CgC*, and analyzed the purine alkaloid profiles of 126 accessions wild *Camellia* germplasm samples belonging to these three species. We found that the profiles of purine alkaloids were highly variable between and within species. Four types of purine alkaloids were detected in *CcC* samples (*n* = 38), three were detected in *Csp* (*n* = 52), and only one was detected in *CgC* (*n* = 36). Theobromine was the primary purine alkaloid in all three species, accounting for over 90% of purine alkaloids in *Csp* and *CcC*, and approximately 50% of purine alkaloids in *CgC*. The high intraspecies variation in the three *Camellia* species examined contrasts the widely cultivated and consumed tea (*Css*), which has a more consistent and predictable purine alkaloid pattern.

Sensory evaluation and preliminary quality composition analysis of green tea products from three *Camellia* species were performed. Notably, none of the three green teas were evaluated to have taste profiles considered that were satisfactory in comparison to commonly consumed *Css* tea. These results provide us with a new sense of understanding why tea (*Css*), rather than other *Camellia* plants (such as, *CcC*, *Csp and CgC*), has ascended over the centuries to its present-day position as a commonly consumed and enjoyed beverage worldwide.

Although the consumption value of the three *Camellia* species investigated in this study may be relatively low, they still represent valuable germplasm resources due to their unique purine alkaloid profiles. Future tea breeding projects may be able to make use of these species as resources for tea variety improvement, or subjects for research on purine alkaloid metabolism. 

In conclusion, this study elucidated the unique purine alkaloid profiles of three *Camellia* species and explored their sensory evaluation characteristics as beverages, which provide a new insight for comprehensive understanding of the relationship between modern cultivated tea varieties and other wild *Camellia* species in components and evolution. It also provided a valuable reference for future developments in tea cultivation and *Camellia* species metabolism research.

## Figures and Tables

**Table 1:** Main floral morphology of three *Camellia* species.

Species	Color and Size	Number of Petals	Styles	Ovary
*C. crassicolumna* Chang	White, 4.8–5.5 cm in diameter	9–12	5-parted, about 2.3 cm in average length, deeply cleft	Spherical, covered with white fluff
*C. gymnogyna* Chang	White, 3.4–4.6 cm in diameter	6–8	3-parted, about 1.9 cm in average length, deeply cleft	Spherical, smooth and glabrous
*C. ptilophylla* Chang	White, 2.3–3.4 cm in diameter	5–8	3-parted, about 1.2 cm in average length, shallow cleft	Spherical, covered with white fluff

**Table 2:** Main morphological characteristics of trees and leaves of three *Camellia* species.

Species	Tree Type	Leaf Size (cm)	Leaf Shape
*C. crassicolumna* Chang	Small trees or arbor, 6–15 m high or higher	(6.8–) 9–(–11.5) 15 × 3(5.5)–6	Oval, oblong, oblong-elliptiptic
*C. ptilophylla* Chang	Shrub or small tree, about 4–7 m high	(7.6–) 8–12 × 3(5.5)–6	Oblong, oblong-elliptiptic
*C. gymnogyna* Chang	Shrub or small tree, about 5–12 m high	86 × 3.6–5	Oval, oblong-elliptiptic

**Table 3:** Variation and distribution of purine alkaloids in three *Camellia* species.

Species		Caffeine	Theobromine	Theophylline	Theacrine	TPA
*CcC* (*n* = 38)	mean ± SD (%)	1.93 ± 0.16 b	1.83 ± 0.14 a	0.07 ± 0.03 b	0.00 ± 0.00 a	3.83 ± 1.29 a
	min (%)	0.00	0.32	0.00	0.00	1.68
	max (%)	3.63	3.18	0.64	0.08	6.37
	median (%)	2.01	1.97	0.01	0.00	3.63
	CV (%)	0.50	0.46	2.28	4.09	0.34
	kurtosis	−0.59	−1.10	4.89	25.65	−0.98
	skewness	−0.24	−0.23	2.40	4.89	0.31
*Csp* (*n* = 52)	mean ± SD (%)	0.15 ± 0.08 a	2.93 ± 0.16 b	0.04 ± 0.01 ab	0.00 ± 0.00 a	3.11 ± 1.58 c
	min (%)	0.00	0.38	0.00	0.00	0.38
	max (%)	3.09	5.25	0.29	0.00	8.63
	median (%)	0.01	2.80	0.03	0.00	2.80
	CV (%)	0.00	0.39	2.29	0.00	0.51
	kurtosis	0.00	−0.42	2.69	0.00	2.86
	skewness	0.00	0.26	2.05	0.00	1.44
*CgC* (*n* = 36)	mean ± SD (%)	0.00 ± 0.00 a	2.19 ± 0.10 a	0.00 ± 0.00 a	0.00 ± 0.00 a	2.19 ± 0.60 b
	min (%)	0.00	0.97	0.00	0.00	0.97
	max (%)	0.00	3.64	0.00	0.00	3.64
	median (%)	0.00	2.28	0.00	0.00	2.28
	CV (%)	0.00	0.40	0.00	0.00	0.27
	kurtosis	0.00	0.40	0.00	0.00	0.39
	skewness	0.00	−0.11	0.00	0.00	−0.11

Note: Values are mean ± SD. Different lowercase letters in the same column mean significant differences at *p* < 0.05 (Tukey’s HSD). CV—coefficient of variation. All units except CV, kurtosis and skewness are percentage of dry weight.

**Table 4:** Principal component analysis of 126 accessions of three *Camellia* species.

Variable	Principle Component		
	PC1	PC2	PC3
Caffeine	0.41	−0.71	−0.30
Theobromine	0.39	0.71	−0.31
Theophylline	0.02	0.01	0.86
Theacrine	0.00	0.00	0.03
Total purine alkaloids	0.82	0.01	0.27
Eigenalue	69.74%	29.89%	0.00
Cumulative	69.74%	99.63%	1.00

**Table 5:** Some special germplasms of three *Camellia* species.

Species	Sample Number	Caffeine	Theobromine	Theophylline	Theacrine
*Csp*	1	3.09 ± 0.01	1.27 ± 0.01	0.00 ± 0.00	0.00 ± 0.00
	40	0.00 ± 0.00	2.55 ± 0.00	0.29 ± 0.00	0.00 ± 0.00
	34	0.00 ± 0.00	3.71 ± 0.01	0.27 ± 0.01	0.00 ± 0.00
	33	0.00 ± 0.00	3.79 ± 0.01	0.26 ± 0.00	0.00 ± 0.00
	37	0.00 ± 0.00	3.15 ± 0.00	0.23 ± 0.01	0.00 ± 0.00
	20	0.02 ± 0.00	2.40 ± 0.01	0.22 ± 0.00	0.00 ± 0.00
	13	0.03 ± 0.00	2.14 ± 0.00	0.19 ± 0.00	0.00 ± 0.00
	26	0.01 ± 0.00	1.93 ± 0.00	0.18 ± 0.00	0.00 ± 0.00
	45	0.04 ± 0.00	2.33 ± 0.00	0.14 ± 0.00	0.00 ± 0.00
	47	0.00 ± 0.00	2.06 ± 0.01	0.13 ± 0.00	0.00 ± 0.00
*CcC*	4	2.13 ± 0.07	2.76 ± 0.54	0.33 ± 0.02	0.03 ± 0.01
	11	3.42 ± 0.03	0.68 ± 0.02	0.00 ± 0.00	0.00 ± 0.00
	5	3.63 ± 0.03	2.51 ± 0.02	0.00 ± 0.00	0.00 ± 0.00
	16	3.02 ± 0.01	0.65 ± 0.02	0.00 ± 0.00	0.00 ± 0.00
	25	2.00 ± 0.05	2.43 ± 0.02	0.13 ± 0.02	0.02 ± 0.01
	23	0.30 ± 0.02	2.53 ± 0.02	0.00 ± 0.00	0.00 ± 0.00
	13	0.01 ± 0.01	2.99 ± 0.02	0.00 ± 0.00	0.00 ± 0.00
	28	0.00 ± 0.00	2.38 ± 0.02	0.51 ± 0.02	0.08 ± 0.01

Note: Values are mean ± SD. All units are percentage of dry weight.

**Table 6:** Contents of catechins, purine alkaloids and total amino acids in three green tea products from three *Camellia* species.

	*Csp*	*CcC*	*CgC*
GC	1.70 ± 0.01 c	0.00 ± 0.00 a	0.79 ± 0.01 b
C	2.25 ± 0.01 c	0.00 ± 0.00 a	0.39 ± 0.01 b
EGC	0.69 ± 0.01 a	1.57 ± 0.01 b	1.73 ± 0.01 c
EGCG	1.81 ± 0.01 c	1.23 ± 0.01 a	1.46 ± 0.01 b
EC	0.64 ± 0.01 b	0.75 ± 0.01 c	0.35 ± 0.01 a
GCG	5.57 ± 0.01 c	0.00 ± 0.00 a	0.02 ± 0.01 b
ECG	0.28 ± 0.01 a	0.46 ± 0.01 c	0.38 ± 0.01 b
CG	0.32 ± 0.01 b	0.00 ± 0.00 a	0.00 ± 0.00 a
EGCG+ECG	2.09 ± 0.02 c	1.69 ± 0.02 a	1.84 ± 0.03 b
EGC+EC	1.33 ± 0.01 a	2.32 ± 0.03 c	2.08 ± 0.01 b
Total Catechins	13.25 ± 0.04c	4.01 ± 0.01 a	5.12 ± 0.01 b
Total Ester-type Catechins	7.98 ± 0.03 c	1.69 ± 0.02 a	1.86 ± 0.03 b
Total Non-ester type Catechins	5.27 ± 0.04 c	2.32 ± 0.03 a	3.26 ± 0.05 b
Percentage of Ester-type catechins in total catechins	60.23%	42.14%	36.33%
Total Amino Acids	1.06 ± 0.03 b	1.31 ± 0.03 c	0.75 ± 0.01 a
Caffeine	0.07 ± 0.01 b	1.56 ± 0.01 c	0.00 ± 0.00 a
Theobromine	3.12 ± 0.01 c	1.62 ± 0.01 a	2.25 ± 0.01 b
Theophylline	0.01 ± 0.00 b	0.03 ± 0.00 c	0.00 ± 0.00 a
Theacrine	0.00 ± 0.00 a	0.00 ± 0.00 a	0.00 ± 0.00 a
Total Purine Alkaloids	3.21 ± 0.02 b	3.21 ± 0.01 b	2.25 ± 0.01a

Note: Values are mean ± SD. Different lowercase letters in the same row mean significant differences at *p* < 0.05 (Tukey’s HSD). C—(+)-catechin; EC—(-)-epicatechin; ECG—(-)-epicatechin gallate; EGC—(-)-epigallocatechin; EGCG—(-)-epigallocatechin gallate. All units are percentage of dry weight.

## Data Availability

The data presented in this study are available on request from the corresponding author.

## Supplementary Materials

The following supporting information can be downloaded at: https://www.mdpi.com/article/10.3390/foods11050627/s1, Figure S1: Schematic diagram of the classification of the tea plants; Figure S2: Peak times of catechins and purine alkaloids; Table S1: Regression Equations, Linear Ranges, and Correlation Coefficients of Eight Catechin Standards; Table S2: Regression Equations, Linear Ranges, and Correlation Coefficients of Four Alkaloids Standards; Table S3: Contents of purine alkaloids in three Camellia species.
