# Supplementary material for: Comparative Analysis of Purine Alkaloids and Main Quality Components of the Three Camellia Species in China
Source: Foods. 2022 Feb 22;11(5):627. doi: 10.3390/foods11050627 (PMC8909160; doi:10.3390/foods11050627)
Supplement: Supplementary file 1 [file foods-11-00627-s001.zip › foods-1578281-supplementary.pdf]

Supporting Data：

Table S1:

Regression Equations, Linear Ranges, and Correlation Coefficients of Eight Catechin Standards

Remain Time/min	Standard	Regression equation	Correlation coefficient
5.592	GC	y=1.50847x-4.10248	0.99855
11.882	EGC	y=2.38286x-1.23412	0.99987
12.122	C	y=7.53476-11.82592	0.99942
17.919	EGCG	y=15.87146x-13.21842	0.99969
20.447	EC	y=7.89751x+13.38833	0.99948
22.664	GCG	y=30.51781x-24.01094	0.99818
27.542	ECG	y=19.61237x+10.05051	0.99968
30.864	CG	y=16.85687x-9.04830	0.99834

Table S2：

Regression Equations, Linear Ranges, and Correlation Coefficients of Four Alkaloids Standards

Remain Time/min	Standard	Regression equation	Correlation coefficient
6.336	theobromine	y=3.31606x-38.15348	0.99755
11.178	theophylline	y=12.30516x+35.30343	0.99993
18.848	theacrine	y=7.53476-11.82592	0.99993
23.708	caffeine	y=15.87146x-13.21842	0.9999

Table S3：

Contents of purine alkaloids in three *Camellia* species

Csp

Number	Caffeine	Theobromine	Theophylline	Theacrine
1	3.09±0.01	1.27±0.01	0.00±0.00	0.00±0.00
2	2.30±0.01	1.49±0.00	0.00±0.00	0.00±0.00
3	1.31±0.01	0.38±0.01	0.00±0.00	0.00±0.00
4	0.33±0.02	4.42±0.00	0.00±0.00	0.00±0.00
5	0.25±0.01	1.95±0.01	0.00±0.00	0.00±0.00
6	0.09±0.01	2.00±0.00	0.00±0.00	0.00±0.00
7	0.04±0.00	3.22±0.01	0.00±0.00	0.00±0.00
8	0.04±0.00	3.09±0.00	0.00±0.00	0.00±0.00
9	0.04±0.01	2.81±0.01	0.00±0.00	0.00±0.00
10	0.03±0.00	5.06±0.01	0.00±0.00	0.00±0.00
11	0.03±0.00	4.33±0.01	0.00±0.00	0.00±0.00
12	0.03±0.01	3.51±0.01	0.00±0.00	0.00±0.00
13	0.03±0.00	2.14±0.00	0.19±0.00	0.00±0.00
14	0.02±0.00	4.39±0.01	0.00±0.00	0.00±0.00
15	0.02±0.00	3.76±0.01	0.00±0.00	0.00±0.00
16	0.02±0.00	3.18±0.00	0.00±0.00	0.00±0.00
17	0.02±0.00	2.83±0.00	0.00±0.00	0.00±0.00
18	0.02±0.00	2.78±0.00	0.00±0.00	0.00±0.00
19	0.02±0.00	2.69±0.00	0.00±0.00	0.00±0.00
20	0.02±0.00	2.40±0.01	0.22±0.00	0.00±0.00
21	0.02±0.00	2.35±0.01	0.00±0.00	0.00±0.00
22	0.02±0.00	1.79±0.00	0.00±0.00	0.00±0.00
23	0.01±0.00	4.89±0.02	0.00±0.00	0.00±0.00
24	0.01±0.00	2.97±0.01	0.00±0.00	0.00±0.00
25	0.01±0.00	2.40±0.01	0.00±0.00	0.00±0.00
26	0.01±0.00	1.93±0.00	0.18±0.00	0.00±0.00
27	0.01±0.00	5.25±0.01	0.00±0.00	0.00±0.00
28	0.01±0.00	4.99±0.01	0.00±0.00	0.00±0.00
29	0.00±0.00	4.90±0.01	0.00±0.00	0.00±0.00
30	0.00±0.00	4.88±0.01	0.00±0.00	0.00±0.00
31	0.00±0.00	3.85±0.00	0.00±0.00	0.00±0.00
32	0.00±0.00	3.83±0.05	0.00±0.00	0.00±0.00
33	0.00±0.00	3.79±0.01	0.26±0.00	0.00±0.00
34	0.00±0.00	3.71±0.01	0.27±0.01	0.00±0.00
35	0.00±0.00	3.67±0.01	0.00±0.00	0.00±0.00
36	0.00±0.00	3.55±0.01	0.00±0.00	0.00±0.00
37	0.00±0.00	3.15±0.00	0.23±0.01	0.00±0.00
38	0.00±0.00	3.12±0.00	0.00±0.00	0.00±0.00
39	0.00±0.00	2.86±0.01	0.00±0.00	0.00±0.00
40	0.00±0.00	2.55±0.00	0.29±0.00	0.00±0.00
41	0.00±0.00	2.48±0.02	0.00±0.00	0.00±0.00
42	0.00±0.00	2.46±0.00	0.00±0.00	0.00±0.00

43	0.00±0.00	2.42±0.00	0.00±0.00	0.00±0.00
44	0.00±0.00	2.40±0.01	0.00±0.00	0.00±0.00
45	0.00±0.00	2.33±0.00	0.14±0.00	0.00±0.00
46	0.00±0.00	2.08±0.01	0.00±0.00	0.00±0.00
47	0.00±0.00	2.06±0.01	0.13±0.00	0.00±0.00
48	0.00±0.00	2.05±0.00	0.00±0.00	0.00±0.00
49	0.00±0.00	1.72±0.00	0.00±0.00	0.00±0.00
50	0.00±0.00	1.70±0.00	0.00±0.00	0.00±0.00
51	0.00±0.00	1.56±0.01	0.00±0.00	0.00±0.00
52	0.00±0.00	0.78±0.01	0.00±0.00	0.00±0.00

CcC

Number	Caffeine	Theobromine	Theophylline	Theacrine
1	2.46±0.07	3.07±0.06	0.00±0.00	0.00±0.00
2	3.60±0.02	2.77±0.05	0.00±0.00	0.00±0.00
3	2.98±0.07	2.17±0.06	0.00±0.00	0.00±0.00
4	2.13±0.07	2.76±0.54	0.33±0.02	0.03±0.01
5	3.63±0.03	2.51±0.02	0.00±0.00	0.00±0.00
6	3.06±0.04	2.09±0.02	0.00±0.00	0.00±0.00
7	2.74±0.05	1.53±0.04	0.00±0.00	0.00±0.00
8	2.31±0.03	2.38±0.01	0.09±0.15	0.00±0.00
9	2.61±0.04	2.15±0.02	0.18±0.15	0.00±0.00
10	1.36±0.03	1.80±0.04	0.00±0.00	0.00±0.00
11	3.42±0.03	0.68±0.02	0.00±0.00	0.00±0.00
12	1.11±0.01	0.57±0.03	0.00±0.00	0.00±0.00
13	0.01±0.01	2.99±0.02	0.00±0.00	0.00±0.00
14	2.75±0.02	3.18±0.03	0.00±0.00	0.00±0.00
15	1.01±0.02	0.49±0.01	0.52±0.03	0.00±0.00
16	3.02±0.01	0.65±0.02	0.00±0.00	0.00±0.00
17	0.61±0.03	2.43±0.02	0.00±0.00	0.00±0.00
18	2.29±0.02	2.91±0.05	0.00±0.00	0.00±0.00
19	0.96±0.02	1.79±0.03	0.00±0.00	0.00±0.00
20	1.74±0.02	2.21±0.02	0.64±0.02	0.00±0.00
21	2.83±0.04	3.04±0.03	0.00±0.00	0.00±0.00
22	1.52±0.02	0.76±0.02	0.00±0.00	0.00±0.00
23	0.30±0.02	2.53±0.02	0.00±0.00	0.00±0.00
24	2.13±0.01	0.96±0.02	0.00±0.00	0.00±0.00
25	2.00±0.05	2.43±0.02	0.13±0.02	0.02±0.01
26	0.59±0.02	1.47±0.02	0.00±0.00	0.00±0.00
27	1.77±0.01	0.32±0.02	0.00±0.00	0.00±0.00
28	0.00±0.00	2.38±0.02	0.51±0.02	0.08±0.01
29	1.90±0.03	1.83±0.02	0.00±0.00	0.00±0.00
30	2.52±0.02	1.06±0.02	0.00±0.00	0.00±0.00
31	1.72±0.01	1.06±0.02	0.00±0.00	0.00±0.00
32	1.26±0.02	1.87±0.02	0.00±0.00	0.00±0.00
33	2.55±0.02	0.97±0.02	0.04±0.01	0.00±0.00
34	2.02±0.01	0.43±0.02	0.00±0.00	0.00±0.00
35	1.43±0.02	1.33±0.02	0.00±0.00	0.00±0.00

36	1.59±0.03	2.09±0.03	0.00±0.00	0.00±0.00
37	0.91±0.02	1.68±0.02	0.31±0.02	0.00±0.00
38	2.39±0.02	2.06±0.02	0.00±0.00	0.00±0.00

CgC

Number	Caffeine	Theobromine	Theophylline	Theacrine
1	0.00±0.00	1.03±0.02	0.00±0.00	0.00±0.00
2	0.00±0.00	2.43±0.02	0.00±0.00	0.00±0.00
3	0.00±0.00	2.34±0.02	0.00±0.00	0.00±0.00
4	0.00±0.00	2.87±0.02	0.00±0.00	0.00±0.00
5	0.00±0.00	2.02±0.01	0.00±0.00	0.00±0.00
6	0.00±0.00	2.31±0.02	0.00±0.00	0.00±0.00
7	0.00±0.00	3.04±0.02	0.00±0.00	0.00±0.00
8	0.00±0.00	3.27±0.02	0.00±0.00	0.00±0.00
9	0.00±0.00	2.61±0.02	0.00±0.00	0.00±0.00
10	0.00±0.00	2.02±0.01	0.00±0.00	0.00±0.00
11	0.00±0.00	1.84±0.02	0.00±0.00	0.00±0.00
12	0.00±0.00	1.54±0.02	0.00±0.00	0.00±0.00
13	0.00±0.00	1.37±0.02	0.00±0.00	0.00±0.00
14	0.00±0.00	1.77±0.02	0.00±0.00	0.00±0.00
15	0.00±0.00	2.51±0.02	0.00±0.00	0.00±0.00
16	0.00±0.00	2.18±0.01	0.00±0.00	0.00±0.00
17	0.00±0.00	0.97±0.01	0.00±0.00	0.00±0.00
18	0.00±0.00	2.22±0.01	0.00±0.00	0.00±0.00
19	0.00±0.00	2.45±0.02	0.00±0.00	0.00±0.00
20	0.00±0.00	2.59±0.29	0.00±0.00	0.00±0.00
21	0.00±0.00	2.52±0.02	0.00±0.00	0.00±0.00
22	0.00±0.00	2.41±0.02	0.00±0.00	0.00±0.00
23	0.00±0.00	1.98±0.02	0.00±0.00	0.00±0.00
24	0.00±0.00	2.12±0.01	0.00±0.00	0.00±0.00
25	0.00±0.00	2.35±0.01	0.00±0.00	0.00±0.00
26	0.00±0.00	2.75±0.01	0.00±0.00	0.00±0.00
27	0.00±0.00	1.93±0.03	0.00±0.00	0.00±0.00
28	0.00±0.00	2.62±0.01	0.00±0.00	0.00±0.00
29	0.00±0.00	2.43±0.02	0.00±0.00	0.00±0.00
30	0.00±0.00	2.33±0.03	0.00±0.00	0.00±0.00
31	0.00±0.00	1.86±0.02	0.00±0.00	0.00±0.00
32	0.00±0.00	2.25±0.01	0.00±0.00	0.00±0.00
33	0.00±0.00	1.88±0.02	0.00±0.00	0.00±0.00
34	0.00±0.00	1.05±0.02	0.00±0.00	0.00±0.00
35	0.00±0.00	1.34±0.02	0.00±0.00	0.00±0.00
36	0.00±0.00	3.64±0.02	0.00±0.00	0.00±0.00

Note: Values are mean ± SD. All units are percentage of dry weight.

Figure S1:

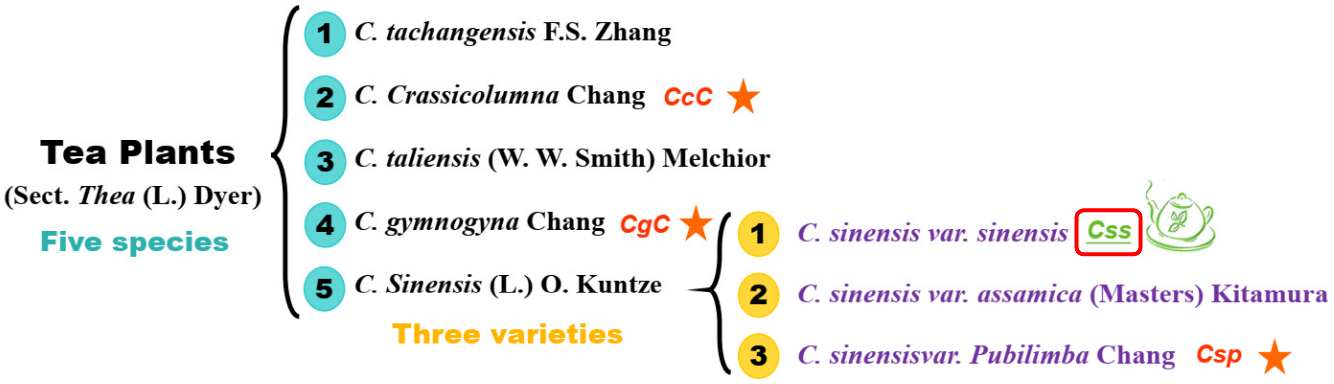

Schematic diagram of the classification of tea plants

The *genus Thea* can be divided into five species, of which *C. Sinensis* (L.) O. Kuntze includes three varieties. ★ : indicates the three species of *Thea* surveyed in the current study. *Css* is the variety commonly cultivated and consumed around the world as tea.

Figure S2:

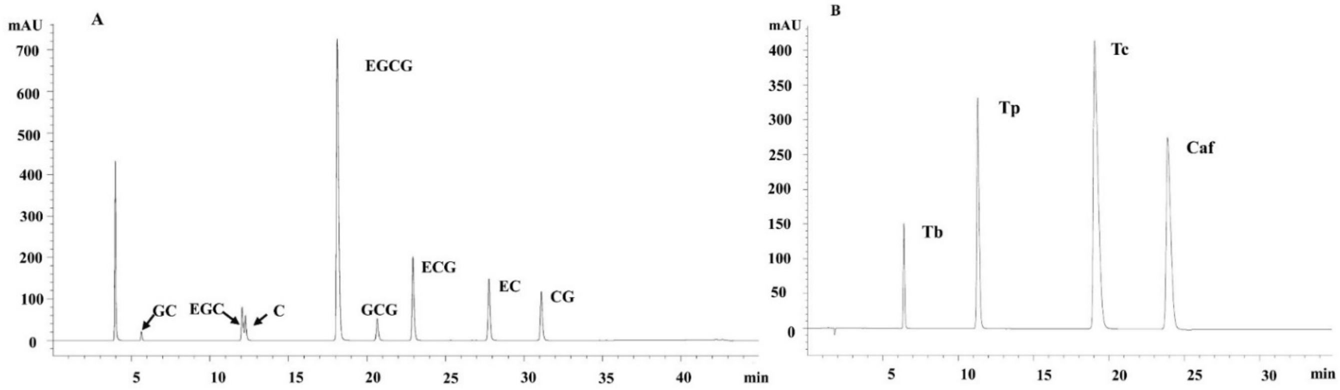

Note: A. Standard for eight catechins; B. Standard for four purine alkaloids.

GC,(+)-gallocatechin; EGC, (-)-epigallocatechin; C, (+)-catechin; EGCG, (-)-epigallocatechin gallate; GCG, (-)-gallocatechin gallate; ECG, (-)-epicatechingallate; EC, (-)-epicatechin; CG, (+)-catechin gallate;Tb, theobromine; Tp, theophylline; Tc, theacrine; Caf, caffeine.
